# The Impact of Role Models on Sexual Minority Women: A Qualitative Interview Study

**DOI:** 10.3390/bs14121119

**Published:** 2024-11-21

**Authors:** Khushi Mann, Salina Tesfamichael, Katharine A. Rimes

**Affiliations:** Department of Psychology, Institute of Psychiatry, Psychology and Neuroscience, King’s College London, London SE5 8AF, UK

**Keywords:** LGBTQ+, sexual minority women, role models, self-esteem

## Abstract

Sexual minority women (e.g., lesbian, bisexual, pansexual) have increased risk of experiencing various mental health problems compared to sexual minority men and heterosexual individuals. Sexual minority women (SMW) have also been found to have lower self-esteem than heterosexual women, which could contribute to poorer mental health. Previous findings suggest that role models could potentially be used to improve LGBTQ+ wellbeing. The current exploratory study investigated SMW’s experiences about the impact of role models or the lack of them and their views about how role models could be used to increase the self-esteem of SMW in potential interventions. Semi-structured qualitative interviews were conducted with 17 SMW. Using thematic analyses, four themes about characteristics of role models were developed: “Similar to me”, “Self-confident about being different”, “Strong and kind”, and “Source of learning and support”. Three themes about the impact of role models were identified: “Increased self-esteem”, “Inspiring personal growth”, and “Lack of role models: I don’t belong”. Content analyses indicated a wide range of ideas about how role models could be used within individual or group self-esteem interventions. Future research could apply these findings to develop or enhance interventions to increase the self-esteem of SMW.

## 1. Introduction

Research has consistently shown that sexual minority women (e.g., lesbian, bisexual, and queer) have an increased rate of several mental health problems and physical risk factors compared to heterosexual men and women. For example, there is evidence that sexual minority women (SMW) experience higher rates of substance use (e.g., tobacco, drugs) and almost every type of alcohol misuse (e.g., alcohol dependence, hazardous drinking) than heterosexual women [1,2,3]. Moreover, SMW are at higher risk of being overweight or obese compared to heterosexual women and report elevated physical health problems such as asthma and cardiovascular diseases [4,5]. SMW have been found to have elevated rates of common mental disorders and low wellbeing compared to heterosexual women [6]. Minority stress theory proposes that the disparities between SMW and heterosexual women’s mental health problems can be explained by stressors unique to identifying as a sexual minority (e.g., stigma, discrimination, victimisation, internalised stigma, expectations of rejection) [7]. Further research has found evidence to suggest that SMW’s increased experiences of heterosexism and trauma experiences (e.g., sexual and physical abuse, witnessing violence) may contribute to SMW’s mental and physical health problems [3,8,9,10]. Sexual minority women experience not only heterosexism but also gender-based prejudice and discrimination that may contribute to the increased risk of common mental health problems relative to men in the general population [11]. For example, there is evidence that daily heterosexism may maintain symptoms of post-traumatic stress disorder in sexual minority women [12].

SMW also have lower self-esteem compared to heterosexual women [13]. There is evidence that minority stressors may contribute to lower self-esteem and self-acceptance in sexual minority individuals compared to heterosexual individuals [14]. Low self-esteem is widely known as a risk factor for a range of mental and physical health problems [15,16,17]. Theory and research have suggested that lower self-esteem could be a mediator in the relationship between minority stress and poorer sexual minority mental health [18]. Therefore, self-esteem has been suggested as a target for intervention to increase wellbeing in sexual minority individuals. There is preliminary evidence of promising results from a self-compassion-focused cognitive behavioural intervention for sexual minority youth.

Theories of positive sexual identity development in sexual minority individuals highlight the importance of social interactions, including with role models (e.g., [19]). In one of the few studies in the area specifically focusing on sexual minority women, Bringaze and White undertook a survey of 262 US lesbian leaders and found that many cited the importance of role models in the development of a positive identity as a lesbian and the coming out process [20]. There is some evidence to support the importance of role models for sexual minority individuals’ general self-esteem too. For example, a qualitative interview study of twenty sexual minority young adults found that role models were often mentioned as important for the development of positive self-esteem.

Few previous research studies have specifically focused on sexual minority women’s views about the important characteristics and self-esteem impact of role models, or whether they felt that role models could be usefully incorporated in self-esteem interventions. This is important given findings from a study with national data from England’s Talking Therapies services (previously known as Improving Access to Psychological Therapies or IAPT services). Rimes et al. [21] found that SMW had higher symptom severity and a risk of a lack of recovery from depression, anxiety, and functional impairment measures after completing psychological interventions compared to their heterosexual counterparts. Foy et al. [22] found that sexual minority adults had a range of challenges and barriers in therapy services such as heterosexism, not feeling understood by their therapist, feeling unable to discuss issues relating to their sexuality, feeling pathologised, and a third were not ‘out’ about their sexuality to their therapist. Similarly, a review concluded that SMW in the UK had worse health outcomes in primary care compared to heterosexual women and faced significant barriers in those services too (e.g., heteronormative assumptions, prejudice from healthcare professionals) [23]. Moreover, a global review found that SMW are distrustful of healthcare providers and show reluctance to engage in important healthcare matters due to fear of mistreatment and prejudice from providers [3]. It is possible that incorporating role models in therapy, or having effective role models outside of healthcare settings, may help sexual minority women to cope with the challenges and barriers in health services.

The current exploratory study aimed to inform the literature by using semi-structured interviews to investigate the characteristics of SMW’s role models. Another key aim was to explore how role models, or the lack of them, had affected the participants, including in relation to their self-esteem or self-acceptance. Finally, participants were asked for their views about how role models could be incorporated into a therapy intervention for SMW with low self-esteem.

## 2. Materials and Methods

### 2.1. Participants

Participants were 17 sexual minority women between the ages of 19 and 55. Participants were included if they were currently living in the UK, were aged above 16 years old, identified as a woman, and identified as having a minority sexual orientation such as lesbian, gay, bisexual, queer, etc. People were not included if they were currently experiencing a serious mental health illness (e.g., bipolar disorder, psychosis, anorexia nervosa, severe depression, etc.) or were considered at a high risk for suicide. This was because taking part in the interview may bring up distressing feelings that could put the participant at a higher mental health risk.

### 2.2. Procedure

Ethical approval for this study was given by the Research Ethics Committee at King’s College London (Ref no.: HR/DP-22/23-33908). Recruitment was undertaken using purposive sampling. The study was advertised primarily online, through social media platforms, online groups, mailing lists, and the Institute of Psychiatry, Psychology and Neuroscience research volunteer circular. Interested participants could sign up to take part through a QR code or through a Qualtrics link [24], which would lead to an introductory page stating the title of the study, the inclusion criteria, and that participation would be compensated by a £15 Amazon voucher. Additionally, there was the information sheet and the option to state interest. Those who provided us with their emails were then contacted to carry out a screening call. This was conducted on Microsoft Teams, and participants were given information about the study and the opportunity to ask questions. They were asked to provide consent to collect preliminary information (a standardised self-esteem questionnaire) and some basic demographic information. Participants then confirmed their interest, completed a digital consent form, and an MS Teams invitation for the interview was sent via email.

Interviews were all held one-to-one online, through Microsoft Teams, and were led by one of two undergraduate students, authors KM and ST, in 90 min timeslots. The interviewers had been trained in qualitative methods on their undergraduate psychology programme, undertook extra reading about interviewing and thematic analysis, and received training and supervision from the senior author. All participants consented to being audio and visually recorded and had a choice to have their camera on or off. The participants were informed that they could take a break or stop the interview at any time and had the right to withdraw their data up until the point when it were analysed. The semi-structured interview schedule was developed within the research team. Feedback about the interview questions was sought from other researchers within the university, including those who identified as sexual minority women and/or who had experiences of low self-esteem, and modified in line with their suggestions. The interview schedule was also slightly adjusted throughout the data collection process to help improve interview flow and content. The interview schedule included questions about the characteristics of role models that they thought were important for both themselves and other sexual minority women. They were asked about the impact of their own role models in childhood, teenage years, adulthood, and about any current role models. Finally, they were asked about whether and how they felt that role models could be incorporated into therapy for sexual minority women wanting help to improve their self-esteem. There were prompts that could be used if needed; therefore, there was not a set number of questions for each participant. At the end, the participants were asked if they had anything further to add that the interview questions had missed, but all indicated that the interview questions had been thorough in eliciting their views about their role models. After the interview, participants were asked how they were feeling and were given the opportunity to talk about their feelings either to the interviewer or to arrange a time to talk to the research supervisor, who is a clinical psychologist with experience working with sexual minority women and people with low self-esteem. However, none of the participants felt the need to talk to the clinical supervisor. Information about support services was provided in the information sheet. The supervisor listened to all the interviews to provide supervision to the interviewers and to inform the data analysis.

### 2.3. Measures

Self-esteem was assessed using the Rosenberg Self-Esteem Scale (RSES) [25]. The RSES is a widely used 10-item questionnaire of global self-esteem. This English-language questionnaire has been widely used in the UK; for example, Bridge et al. [26] found that it had acceptable internal consistency and was sufficiently sensitive to be used in participant eligibility for a low self-esteem intervention study with sexual minority participants. Statements range on a 4-point Likert scale, from ‘Strongly agree’ to ‘Strongly disagree’. Scores are totalled and can range from 0 to 30, with higher scores indicating higher self-esteem.

### 2.4. Data Analysis

Each automatic interview transcript from MS Teams was proof-read, anonymised, and corrected to match the audio recordings verbatim by one of the two interviewers. Analysis of participants’ experiences, feelings, and ideas about role models followed Braun and Clarke’s six-step thematic analysis guidelines [27]. The first author read all the transcripts to further familiarise herself with the data (step 1). Next, initial codes were created (step 2) and organised into potential themes and subthemes (step 3). Themes were then reviewed, refined, and names discussed over a number of sessions with the research supervisor (steps 4 and 5). When the first and senior author had finalised their proposed themes, they were sent to the second interviewer to check whether they were appropriate. The first author undertook the first draft of the report (step 6). Throughout the interview and analysis processes, a reflexive practice journal was kept to ensure high-quality standards for qualitative research. The interviewers received regular supervision from the senior researcher in which the data and possible themes were discussed so that researcher triangulation could take place.

Data on types of role models, ideas on how to incorporate role models into a therapy intervention, and other ideas on how to use role models to increase the self-esteem of sexual minority women were analysed using a combination of Hsieh and Shannon’s conventional and summative content analysis methods [28]. Firstly, conventional content analysis was used to create codes directly from the data, independently from the interview schedule, to summarise the different types of role models. Additionally, summative content analysis was used to summarise participants’ ideas and opinions on including role models in therapy interventions for sexual minority women with lower self-esteem.

## 3. Results

### 3.1. Participant Characteristics

The mean age of the participants was 31 (SD = 11), with a range of 19–55. Other participant characteristics and self-esteem scores are summarised in Table 1. In the following results sections, quotes are labelled with the associated age, gender (W = woman, NB = non-binary), self-esteem score, ethnicity (WB = white British, WW = white Welsh, WE = white European, WM = white mixed, MWM = mixed white Mexican, MBC = mixed black Caribbean, BI = British Asian, A = Asian), and participant number.

### 3.2. Types of Role Models

Content analysis indicated a wide range of different types of role models (Table 2). For most participants, family members and teachers were their main role models in childhood, with many particularly pointing out a female majority amongst them (e.g., mothers, grandmothers, aunts). However, about half of the participants mentioned their fathers. This was often due to the emotional comfort they felt from them and their ability to relate to their interests. The most common form of role models were friends (e.g., from school, university, and groups), with several naming female and/or sexual minority individuals. A large category of role models were celebrities, or people in the media, that participants did not know personally. A variety of role models included those within the same career field and characters from books and TV shows.

### 3.3. Characteristics of Role Models

**Figure 1:**
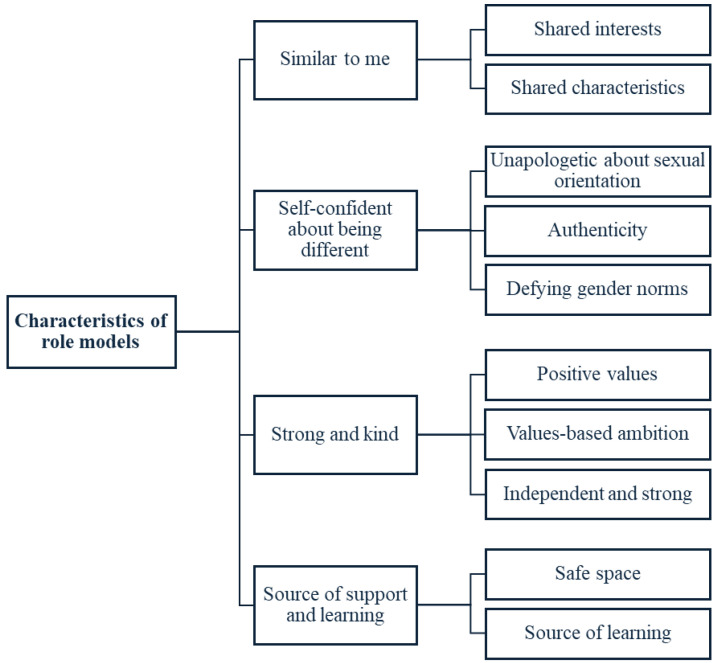
Diagram showing four themes about the characteristics of role models and their sub-themes.

Using thematic analysis, four key inter-related themes about the characteristics of the participants’ role models were developed. These were ‘Similar to me’, ‘Self-confident about being different’, ‘Strong and Kind’, and ‘Source of support and learning’. The sub-themes within these overarching themes are outlined below and summarised in Figure 1.

#### 3.3.1. Theme 1: Similar to Me

##### Shared Interests

Many participants mentioned sharing the same interests as their role models. Several mentioned looking up to people within the same career field as themselves, which were all science, technology, engineering, and mathematic (STEM)-types of work. For example, one participant said that most of their childhood role models were “people that were doing similar kind of sciency subjects” (29WB23WM12) to themself. Another participant looked up to “high up women, in the stem field”, specifically pointing out the lack of women in the industry (24WP11WB2).

Another commonly shared interest was sports. Many participants reported feeling inspired by successful LGBTQ+ women in the industry, “a woman, who is gay, got what is seen as the best sports prize” (23WL19WB10). They also noted the importance of these role models in normalising exposure to queer people, mentioning that people who played sports were less afraid to come out compared to others, “which means it’s opening opportunities for there to be role models for younger people who are gay”. Some participants also looked up to fellow sports team members as their role models, noting “so many queer people” amongst them (26WQ14WB5).

##### Shared Characteristics

All participants had at least one role model with shared characteristics to themselves. All participants named people with minority sexual orientations, such as in the media (e.g., Lil Nas X, Ruby Rose) or within their family (e.g., aunts, cousins). Some participants had in childhood looked up to role models who were LGBTQ+, although not with conscious intention at the time. Upon reflection, one noted that their role model in school was “visibly queer” (25NBQ11MWM15). Another mentioned she was “searching out media to just see queer women” before coming to realise her own sexuality (26WQ14WB5). For participants who did not have LGBT+ role models in person, they often found them in the media. One participant described the importance of having role models in the media “before the internet became accessible to me… it was more difficult to sort of find role models” (20WL19WE7). Another participant who did not have LGBTQ+ role models in real life resorted to making heterosexual TV characters “queer in our minds”, in a way of “aligning myself with queer community and Queer icons” (25NBQ11MWM15).

Most participants looked up to role models with a combination of shared characteristics, such as people from various minority groups. One participant spoke fondly of role models with whom they shared intersectional identities, saying they look up to role models who “are really affirming of black people with disabilities” (44WQ17MBC8), whilst another participant looked up to a “gay non-binary” reverend (20WL19WE7). Similarly, some participants spoke about the importance of having an “exposure to a really diverse range of people” (28WB23WB11) to form role models. For example, one said that without diversity, there is no ability to relate and resonate with a role model and “the higher up you go, the narrower it becomes and much more predominantly white and higher socioeconomic status” (29WB23WM12).

#### 3.3.2. Theme 2: Self-Confident About Being Different

##### Unapologetic About Sexual Orientation

Participants often spoke about having role models who confidently express their sexual orientation. One participant mentioned looking up to lesbian fictional characters and admiring their popularity despite them being “so unapologetic about who they are, and they’re so outside of these traditional roles”. They also stated the importance of role models being “really confident expressing their sexuality”, pointing out that being unapologetic was something they also “really wanted to be” (20WL19WE7). Similarly, some looked up to their partners, with one participant admiring their confidence and security in their identity despite the heterosexism in society (28WB23WB11). Another participant spoke about the status of LGBTQ+ people in society, saying that they often must give explanations for identifying differently in order to be accepted: “there is a perception that queer people owe their stories to be able to be respected”. They said that their role models are “unashamedly themselves” and should identify confidently without feeling the need to justify: “it’s good to stand your ground saying look this is how I identify and I am not here to prove to you that this is who I am and I don’t have to tell you any more than I want to” (26WQ14WB5).

##### Authenticity

Several participants mentioned authenticity as a key characteristic they look up to in a role model. One participant described that an ideal LGBTQ+ role model should have a “realistic lifestyle”, being confident in themselves and acknowledging and accepting their own flaws, “have imperfections and be okay with them… not trying to be overly positive about them” (20WB13A1). Similarly, there was emphasis on the need for queer role models to have ‘normal’ integration within society, not having their sexual orientation define what they do for a living, or how they live their lives. Other participants spoke about the importance of LGBTQ+ models being authentic and sharing experiences to create accurate and healthy standards for individuals to look up to and relate to.

“It’s also not right to assume someone’s perfect cause it makes you feel like you’re less than, and you’re kind of striving for something that isn’t real” (19WB15BI6).

##### Defying Gender Norms

Another common characteristic of role models was deviating from the norm. For some participants, this was in the form of fictional characters, such as females “breaking gender boundaries” and pursuing skills, showing traits of strength and independence (25NBQ11MWM15). Lesbian characters on TV and movies were mentioned, specifically those who were butch-presenting (e.g., from the ‘L-Word’), being admired for their appearances, “completely reshaped the narrative of what is attractiveness” (20WL19WE7). Other participants named women in male-dominated career fields as their role models, such as “lesbian scientists” and political figures (55WL15WE4; 20WL19WE7; 50WG21WW13). Several participants looked up to women in STEM fields, describing them as ambitious, empowering, and strong, highlighting the under-representation of SMW, and women in general, in STEM. Another participant pointed out the necessity for these role models in medicine, pointing out the power difference in this field (37WP21WB14).

Some participants noted their fathers as their only male role models, often picking out and looking up to characteristics of them that were gender nonconforming. For example, one participant valued their father’s “feminine masculine traits”, describing him as “very empathetic” (24WP11WB2), whilst another labelled their father as “emotionally pragmatic” (28WB24WB16).

#### 3.3.3. Theme 3: Strong and Kind

##### Positive Values

Many described their role models as having a range of positive values. One participant specifically looked up to their father from a “values point of view”, labelling him as charitable and empathetic towards others (24WP11WB2). Another participant described their role models as solely content and positive, “happy and gay” (55WL15WE4).

Being body positive was also a key characteristic that several participants pointed out for their role models. For example, “celebrating cellulite and stretch marks” was described as essential, with this participant pointing out the large role that body image plays on the self-image of SMW: “body dysmorphia plays such as a huge role in how people perceive themselves and then what they’re striving for” (29WB23WM12).

##### Values-Based Ambition

Several participants looked up to role models that showed ambition and success, most of which were career-oriented. Some spoke about their childhood role models being “determined” in their careers, such as sports (20WB13A1), and being a “hard worker, and somebody who will just keep going” towards their goals (26WQ14WB5). One participant admired her fathers “sense of achievement” in his career, portraying his passion for what he is interested in as inspiring and true to oneself:

“Doing what you think matters rather than what gets you the most prestige or the most money, so that was really important, and doing what makes you happy” (24WP11WB2).

##### Independent and Strong

Many participants said their role models were “strong in their opinions” (19WB15BI6), describing them as firm and not swaying towards societal norms. Others spoke about looking up to female fictional characters, those who were smart, “thinking outside the box” (25NBQ11MWM15), and “super strong, out and about kicking crime” (31WP15WE9). Another participant mentioned looking up to famous sports women, admiring them for not being “girly,” and staying true to themselves:

“They were capable, they had muscles, they were good at what they did, and it was kind of all the things that I thought was amazing and that I wanted to be” (50WG21WW13).

Another participant reflected on growing up in a time when “‘feminist’ was a dirty word”, mentioning she looked up to females who deviated from those norms, striving for independence and breaking boundaries, “she’s so strong and so brave… she had charisma”, showing that “you can be in control of your life” as a woman (55WL15WE4). Similarly, other participants saw people who were vocal about social issues as their role models “very strong voices… quite political strong messages of angst” (43NBG16WB17). Another spoke about the importance of starting conversations, saying people who start “public discourse”, by bringing light to topics such as body positivity and LGBTQ+ issues, can be seen as “leaders of society” and ideal role models for young queer people (20WL19WE7).

#### 3.3.4. Theme 4: Source of Support and Learning

##### Safe Space

Most participants had at least one role model who acted as a safe space, such as family members and teachers, forming the foundations of childhood role models. Some described their family members as “warm” and “nurturing” (24WP11WB2). Other participants portrayed their parental figures as reassuring and supportive, acting as references in times of difficulty and distress. For example, one participant admired their mothers’ “emotional intelligence”, exuding happiness and making them feel comforted (28WB23WB11). For many participants, this was their grandma. For example, one mentioned that their grandmother was “very supportive”, and they also thought about her when not in her presence: “I was quite nervous. And then I remembered that my grandma wouldn’t be nervous” (23WL19WB10). Similarly, a participant named their grandmother as their “biggest role model”, being closer with her on an “emotional level” than their mother (26WQ14WB5). Several participants also looked up to their fathers in childhood, saying they would turn to their father in similar situations, “he always knew what to do, always knew the answer” (43NBG16WB17).

##### Source of Learning

For many, role models acted as a source of learning. For example, growing up, some participants looked up to family members who provided guidance when “academically struggling at school” (23WL19WB10). Some participants utilised role models to explore and learn about their own sexual identity. For example, one reported looking up to queer actresses, saying the exposure helped her “learn more” about her identity (20WB13A1). Another emphasised the need for role models to be educational about identity, specifying that they are crucial when an individual is going through the coming out process, to answer questions which would reassure the individual: “when you first come out, you have a lot of doubt and a lot of worry and a lot of concerns about what everyone’s gonna think” (23WL19WB10). When referencing their own experiences, one participant described the importance of having educative role models as a way of supporting individuals in discovering and understanding their own identity:

“As a queer person, my education has been terrible. I’ve had to learn everything myself, and the things I just didn’t know because there was no one to teach me” (44WQ17MBC8).

### 3.4. Impact of Role Models

**Figure 2:**
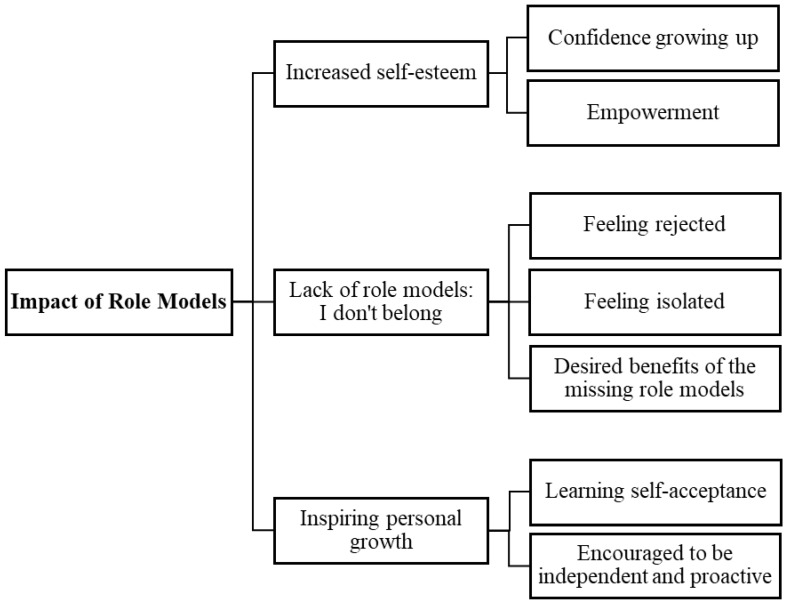
Diagram showing the three themes about the impact of role models and their sub-themes.

Three key inter-related themes about the impact of the participants’ role models were developed from the thematic analysis. These were ‘Increased self-esteem’, ‘Lack of role models: I don’t belong’, and ‘Inspiring personal growth’. The subthemes within each of these overarching themes are described below and in Figure 2.

#### 3.4.1. Theme 1: Increased Self-Esteem

##### Confidence Growing up

Several participants highlighted the confidence boost that teachers provided for them. One participant mentioned the deep value of teachers’ academic validation: “she was the one of the only people that believed in me” (23WL19WB10). Another participant commented on the presence of “strong feminist” female teachers that helped them learn that “women can do anything that men can” (25NBQ11MWM15), enabling them to feel validated and self-confident. Participants not only looked up to female teachers but also older female students in school. One noted the confidence of her friend to “stand up for herself” against things such as homophobic comments, giving her courage to do the same (20WB13A1). Another noted the impact of just seeing older students express themselves freely around their peers, describing it as encouraging.

“Being able to be who she wants to be at school. I was so worried about doing that or expressing myself in a way that felt authentic… I was impressed that she was. So yeah, she had a big role” (24WP11WB2).

Many participants reflected positively on having role models similar to them during childhood or adolescence. Some spoke about looking up to influential pop musicians, those with distinct dress styles such as rock bands and alternative female artists. One participant mentioned the large contribution they made in her everyday life “defined the clothes that I wore, the attitude that I had, the way that I spoke, the things that I was into, everything”. They also described feeling “validated, understood, seen” and “empowered” (43NBG16WB17) seeing them dress in way that was different and self-expressive.

One participant spoke about the personal benefits of having LGBTQ+ exposure in the media, stating that exposure to queer actresses enabled her to overcome “confusion” about her own identity (20WB13A1). Similarly, another participant spoke about the impact of having a family member in a lesbian relationship, describing the feelings of security and confidence it gave her “I’ve always felt very comfortable in my sexuality around that side of my family” (29WB23WM12).

##### Empowerment

Participants often spoke about feeling empowered by their role models, particularly when gaining LGBTQ+ role models in their later life, after having a lack of them in childhood. Many participants talked about the impact of meeting and being around gay women in real life for the first time, for example “I felt liberated” (55WL15WE4) and “I felt empowered to be whoever I wanted to be” (25NBQ11MWM15).

Many described looking up to queer people on social media, identifying with their struggles and experiences and adapting their values (26WQ14WB5, 19WB15BI6, 23WL19WB10, 25NBQ11MWM15). Several participants talked about the important effects of gaining role models with shared characteristics to them. One participant talked about the relief of finally having a black role model, someone who could understand and listen to their experiences and struggles of being black and queer: “it’s like being able to breathe again… Having a black lecturer at university and having him understand how much you lose when you come out as a black queer person, was just amazing. Like I’ve never been able to explain it to anybody else. And he understood.” (44WQ17MBC8). Another spoke about meeting LGBTQ+ religious figures for the first time, allowing her to “reconcile” with her religion and feel accepted by the community (20WL19WE7).

A couple of participants spoke about growing up during the time of the Section 28 legislative, which prohibited the ‘promotion’ of homosexuality by local authorities and schools. One said that whilst the legislation itself was “horrifying and shocking”, going to protests and anti-homophobic marches allowed her to integrate with people with similar experiences, saying she “found an identity and started to be able to relate to other people” (50WG21WW13). Similarly, another participant said all the LGBT+ individuals she met at the time were her role models as a “collective”, saying it was the first time she was surrounded by “happy lesbians”: “it really set me free” (55WL15WE4). Moreover, she described it as a “revelation”, realising she could also be like the people she was newly surrounded by.

#### 3.4.2. Theme 2: Lack of Role Models: I Don’t Belong

##### Feeling Rejected

Many participants who grew up without the presence of role models often described feelings of rejection and being left out. For example, one spoke about the lack of positive LGBT+ people in society growing up, erasing possibilities for them to question and explore their sexuality and having to follow the restricted norm: “I discounted a huge part of who I am in order to fit in with the role models that I was given, which were not who I could identify with, so it was very, very difficult. It caused 40 years of confusion and unhappiness” (43NBG16WB17).

Some spoke about being rejected by their religious community growing up, saying that as they came out, their friends “disappeared”, making them feel “very sad” (23WL19WB10). The participant developed internalised homophobia, resulting in trying to change and become like the people around them: “everyone around me disagrees with it, so therefore I should probably try and be like everyone else” (23WL19WB10). Another participant described how coming from a community with anti-LGBTQ+ views led her to feel that “this religion is against me” (20WL19WE7).

Some participants spoke about not having any positive role models to relate to in terms of race and ethnicity. One participant pointed out the discrepancies between the representation of queer role models on social media and their own experiences, saying those who are “not accepted at home” find it a lot harder to relate to and feel out of reach: “influencers these days, they have the support, their families, they have loads of friends, and they have what’s called ‘chosen families’… but that doesn’t seem incredibly attainable for me” (19WL15BI3). Another participant pointed out that all the queer people around them growing up were white, noting that at all potential opportunities to have them as role models, “they’d say something racist” (44WQ17MBC8). This resulted in pessimism and feeling let down, and eventually a fear of role models as a whole: “I’m quite scared of having role models because it’s like, you don’t want to invest too much in them, because they’re human, just like the rest of us, and they’re gonna mess up. So, if you put all your investment in being like them, you’re going to find that that was fake at some point, so don’t bother” (44WQ17MBC8).

##### Feeling Isolated

Many participants described feeling alone due to a lack of role models and how this affected them. For example, one participant mentioned how listening to people’s experiences of discrimination helped them overcome and reflect on their own trauma, “if I’d had that growing up, I wouldn’t have felt so alone” (44WQ17MBC8). Another participant said they “didn’t know anyone who felt the way I felt”, leading to internalised homophobia, “I’m disgusting”, and shutting away from society, becoming “terminally online” (25NBQ11MWM15). Inaccessibility to role models of colour who were also a sexual minority often related to feeling isolated. One participant said, “there was nobody who lived a good life, but I could look up to who was queer and Black”. They described the considerable impact it had on finding their own identity, “I was very lost for a very long time… I didn’t feel there was anybody who was enough like me that I could use them as a pathfinder” (44WQ17MBC8). They also reported the inability to see people unlike themself as a role model, such as white people, due to their lack of knowledge and acceptance of people of colour: “I can’t look up to any of these people really, because they know nothing about me. I’m kind of an intruder in the system” (44WQ17MBC8).

##### Desired Benefits of the Missing Role Models

Many participants spoke about how they could have potentially benefitted from the role models who they lacked. Several participants felt that if they had religious queer role models growing up, they would have come to terms with their sexuality sooner, without having gone through issues such as internalised homophobia. They stated the large role religion played in their lives and wished for some exposure: “if I had more role models earlier on, in the Christian context, which was a very big part of my upbringing, I would’ve explored it sooner” (28WB24WB16). Another participant mentioned the lack of “older queer people of ethnic minorities”, stating it would have been beneficial to relate to and gain a “positive outlook on my life” (19WL15BI3). Similarly, another participant noted, “It’s nice to have more representation for women and people of colour and queer people, and there just isn’t any… in your career or whatever it is-anything that you aspire to be, makes a huge difference and seeing someone that you kind of identify with makes you think ‘Oh no, there is space for me there’” (29WB23WM12). Another said that most role models for SMW identified as lesbian and underlined the need for “more education and less stigmatisation, and fetishization of bisexuality” (29WB23WM12).

#### 3.4.3. Theme 3: Inspiring Personal Growth

##### Learning Self-Acceptance

Participants reflected on how role models supported the development of self-acceptance. For example, some mentioned seeing characters on television for the first time, who were outside of the traditional norms, such as those who were unapologetic about their sexuality and gender nonconformity, helping them realise “it’s okay to be different”. (20WL19WE7). Another participant learnt self-acceptance by mirroring their role models’ values, using their “thoughts or ethos they take into life, and trying to replicate that in myself” (28WB23WB11). Some learnt how to overcome internalised homophobia through connections of trauma and coping mechanisms, watching “how they dealt with some of the prejudice and then using their strategy for myself” (44WQ17MBC8).

##### Encouraged to Be Independent and Proactive

Several participants described feeling motivated by their role models, such as to be proactive and independent about making decisions. For many, this came from teachers. For example, one participant was thankful for their tutor for believing in them, describing how they encouraged them to reflect and utilise their strengths, making them feel that “I did actually have a talent, and I was actually good” (43NBG16WB17). Another participant reflected on how they observed their role models and then adapted ways to learn about themselves and integrate with the surrounding environments, “how do they build community, how do they deal with prejudice, how are they inclusive, how do you build chosen family?” (44WQ17MBC8).

A handful of participants illustrated a personal development trajectory in how they utilised role models. Some described less of a need for them over time. Others spoke about changing from wanting to become like their role models to learning through their experiences. Some reflected on idolising role models: “I’m a bit more careful about who I’m idolizing. I realised that people aren’t perfect and that you can idolize a certain part of somebody, but you can’t necessarily try to become them. And I think that’s something that I kind of let go of overtime” (19WL15BI3).

### 3.5. Role Models in Therapy

Participants gave various suggestions on how to incorporate the usage of role models into a self-esteem therapy intervention for SMW (Table 3). Many participants recommended a reflective strategy, similar to that of the interview style, asking individuals to think back about their own role models. Some stated that they did not realise that they saw people as role models until they had the opportunity to reflect in the interview setting. For those without access or exposure to role models, forming tailored lists of potential people was suggested. These could include fictional characters, celebrities, and/or people personally known to the individual, specifically role models who are diverse and, preferably, LGBTQ+-identifying. Consequently, participants advised using their past and current role models as a template to relate to and learn from by comparing their attributes and experiences and setting suitable goals to ultimately increase self-esteem.

However, many noted potential disadvantages to this approach, such as the potential to feel inferior to role models or an unhealthy “comparative environment” (23WL19WB10), where individuals are left unhappy from thinking that they will never be like or become their role model. Another commonly mentioned limitation of including role models in the therapy intervention is that individuals may fixate on and over-attach to a role model. Some mentioned the possibility of losing themselves by “forging their own path” (44WQ17MBC8) by trying to copy their role models instead of using them as a guide for self-development.

Participants offered ways to overcome the listed limitations. For example, some recommended utilising several role models at once, to prevent fixating on one specific model, allowing the individual to “pick and choose” different characteristics and experiences (19WL15BI3; 19WB15BI6). Some participants spoke about the possibility of using role models with a negative past, such as celebrities who may have been ‘toxic’ in the media. They highlighted the importance of being transparent, such as exploring the models’ life history, and explaining that “role models are not perfect” (19WB15BI6).

Some participants offered a preference of group therapy over one-to-one therapy. This was proposed as an idea for individuals to become each other’s role models by sharing their feelings and experiences.

Participants also recommended places for individuals to find role models outside of the therapy setting (Table 4). Several participants said schools are the best place, such as in LGBTQ+-friendly clubs, and assemblies, labelling these crucial, as the first LGBTQ+ exposure that many young SMW will have. The most recommended source was online, on social media, with participants naming sites such as YouTube and Instagram as ideal for SMW to broaden their diversity of role models.

## 4. Discussion

This exploratory qualitative interview study with sexual minority women examined the characteristics and impact of their role models and how they thought that role models could be used in a self-esteem therapy intervention.

The finding that it was important for role models to be similar in some way to the individual is consistent with Tajfel’s Social Identity Theory [29], which suggests that membership to a specific social group supports self-esteem. Being able to relate to similar role models had a range of benefits for the participants, including enhancing self-acceptance, self-esteem, self-identity development and confidence, as well as being a source of learning and, if known in real life, support too. Conversely, the lack of such models was noted as impeding self-acceptance and development of a positive identity as a sexual minority individual. These findings are consistent with theories of positive sexual identity development which highlight the importance of social contact with other sexual minority people [19].

The participants highlighted the lack of representation of role models with other intersectional identities, describing similarities in race, ethnicity, religion, gender, disability, and weight as important factors in role models for SMW. Under-representation of these identities added to SMW’s feelings of isolation, rejection, and pessimism. This was particularly the case for those who were from ethnic and racial minorities, often not being able to look up to white LGBTQ+ people due to a lack of relatable experiences and understanding. This is in line with previous findings that LGBTQ+ people of colour are marginalised in multiple ways and experience additional stressors unique to race and ethnicity (e.g., microaggressions, racial discrimination), leading to further negative health outcomes [30,31].

Many SMW, especially those who did not have access to familial role models or in their real-life environments, saw media role models as a source of informal learning and supported them to come to terms with their sexuality sooner. This confirms previous findings that social media can be a key source for LGBTQ+ individuals [32]. Whilst current portrayals of LGBTQ+ people on television are progressing, SMW characters are still under-represented and tend to have toxic portrayals compared to sexual minority men on television [33,34]. Previous research also found that exposures to such inaccessible role models were associated with increased psychological distress in LGBT Youth [35]. Instead, participants preferred sexual minority role models who were authentic and integrated in society with ‘realistic lifestyles’, including influencers on platforms such as YouTube and Instagram. It has previously been noted that lesbian content creators who share their daily life online could reflect the everyday experiences of LGBTQ+ individuals more accurately than characters on TV [36]. Previous US research using online surveys also found that exposure to LGBTQ+ YouTubers and other influencers may increase the self-esteem of sexual minority individuals [37,38].

Role models being confident about their sexual identity was not the only characteristic important to participants. For example, many SMW had these role models within the same occupation as themself, especially in STEM careers (science, technology, engineering, and mathematics) or other professions that tend to be male-dominated. Previous findings have also shed light on the lack of visible LGBTQ+ role models in STEM and further showed major problems facing sexual minority individuals in these professions, promoting gender role stereotypes [39]. For these reasons, it may be useful to encourage SMW to find role models within their career field.

Personal qualities most valued in role models included being strong and independent, hard-working while also being authentic, kind, and having empathy. Future research could investigate whether these personal characteristics, and the above-noted gender nonconformity, are also reported as being important in the role models of heterosexual women or are specific to sexual minority women. Similarly, researchers could compare these findings with those for sexual minority men. The current findings could be used to construct a survey to investigate whether the results are replicated with a larger UK sample, and comparisons could also be made cross-nationally.

### 4.1. Clinical Implications

The large impact that role models have had on these participants, and their positive views about the use of role models to support self-esteem, indicates that increasing access to and use of role models could play a helpful role in improving the mental health of SMW. Considering the barriers that SMW can face when trying to find role models (e.g., fear of being outed, not enough representation), tools may be helpful, such as lists of celebrity role models, or reading lists of books containing inspirational characters. Examples of role models are recommended to include both sexual minority women and people who identify with other minoritised backgrounds similar to the individual; intersectional characteristics matching the individual would be even better. The current findings pointed to the importance of role models in general in childhood, particularly gender nonconforming girls and women, so this could be considered a general self-esteem support intervention in schools. However, gaining sexual minority role models in adolescence and adulthood also helped improve participants’ mental wellbeing, allowing SMW to feel empowered and confident, increasing self-esteem. Some participants urged caution in recommending role models to SMW in a therapy intervention to increase self-esteem. For example, some individuals may compare themselves unfavourably to these individuals. The development of interventions using role models should be developed collaboratively with SMW with low self-esteem. Although most participants would prefer individual therapy, some also expressed interest in group therapy, and there were also suggestions that SMW can become useful role models for one another in this setting.

### 4.2. Limitations

The current study has several limitations. Firstly, it is likely that participants’ responses, as with any interview design, will not have fully reflected their experiences, thoughts, and feelings. For example, as noted with the role models themselves, perceived similarity between the participant and the interviewer may have influenced what the participants disclosed or other perceptions of the participant about what felt safe or appropriate to talk about. Some participants noted that they had not thought about the issue before and needed time to remember and think through the questions asked; a follow-up session might have been helpful to support this. Furthermore, only seventeen participants were recruited and there were biases in the sampling, so caution is required in drawing conclusions. Whilst efforts were made to recruit a diverse sample, the majority of participants identified as white British, or came from other white ethnic backgrounds. It is especially important to diversify research, as SMW of colour were found to have additional factors influencing their self-esteem and more difficulty in identifying appropriate role models. In addition, all participants were either undertaking or had completed a university degree, so further research needs to focus on recruiting participants with different educational experiences, who may have different views and experiences of role models. Although the study participants ranged in age from young adulthood to midlife, efforts to recruit older sexual minority women were not successful. Older sexual minority women are likely to have had very different experiences of role models as they had grown up in a social context that included homosexuality being illegal and considered to be a mental illness. Another limitation is that it is possible that people with previous experience of mental health problems were more attracted to study participation. A future study may wish to recruit a sample with no previous experience of mental health problems to investigate whether similar themes were identified. Furthermore, the participants recruited by social media may be particularly likely to report social media role models, so it cannot be assumed that the results could be used to generalise to other participants who do not use social media. Future studies should aim to recruit larger sample sizes with participants with a broader range of characteristics. Another limitation was that due to time constraints, it was not possible to seek participant feedback about the themes identified. This is recommended for future studies to help ensure the trustworthiness of the analysis. 

## 5. Conclusions

This exploratory study reported the positive impact role models had on participants, providing preliminary evidence for the importance of them for sexual minority women. The findings also suggested that psychological interventions, including the use of role models, may be useful in improving the self-esteem in this group. Further research should seek to test and develop interventions, including role models. One type of potential intervention is an individual compassion-focused CBT intervention, such as the one developed by researchers at King’s College London [29]. This was recently modified specifically for sexual minority women, with promising results [40]. It has a modular format which could easily incorporate a module on using role models for participants who express an interest. Participants in the present study also felt that role models could be incorporated into group interventions, where group members could even act as role models for each other. In both individual and group interventions, participants could be introduced to different ways of accessing role models, both for people with whom they come into contact in everyday life (e.g., friends, family, work colleagues, members of hobby groups or religious organisations) as well as via the media and social media. It was noted that care should be taken to ensure that sexual minority women with low self-esteem do not compare themselves unfavourably to role models but instead use them as a source of support, connection, validation, self-acceptance, inspiration, and empowerment.

## Figures and Tables

**Table 1:** Participant demographics and self-esteem scores.

Variable	*N*	%
Sexuality		
Gay woman	2	12
Lesbian	4	24
Bisexual	5	29
Queer	3	18
Pansexual	3	18
Gender Identity		
Woman	15	88
Non-binary	2	12
Ethnicity		
White British	7	41
White Welsh	1	6
White European	3	18
White mixed	1	6
Mixed white, Mexican	1	6
Mixed black Caribbean	1	6
British Indian	2	12
Asian	1	6
UK Residence		
Greater London	13	76
Wales	1	6
Northen England	3	18
Education level		
Undertaking undergraduate degree	4	24
Completed undergraduate degree	13	76
Employment status		
Student	6	35
Employed	11	65
Measures	Mean (SD)	Range
Rosenberg Self-Esteem Scale score	17 (4)	(11–24)

**Table 2:** Types of role models, with gender and sexual orientation where specified.

		N/S	F	M	GMi	N/S	L/GW	GM	B/P	Q	H
Family	N (%)	%	%	%	%	%	%	%	%	%	%
Mother	7 (41)	-	100	-	-	100	-	-	-	-	-
Father	8 (47)	-	-	100	-	100	-	-	-	-	-
Older sister	3 (18)	-	100	-	-	100	-	-	-	-	-
Grandmother	7 (41)	-	100	-	-	100	-	-	-	-	-
Grandfather	1 (6)	-	-	100	-	100	-	-	-	-	-
Aunt	5 (29)	-	80	-	20	80	20				
Uncle	1 (6)	-	-	100	-	100	-	-	-	-	-
Cousin	1 (6)	-	100	-	-	-	100	-	-	-	-
Others known personally											
Partner	4 (24)	-	75	25	-	75	25	-	-	-	-
LGBT friends	8 (47)	64	18	18	-	9	45	18	9	18	-
Other friends	11 (65)	55	45	-	-	100	-	-	-	-	-
Family friends	2 (12)	-	50	50	-	50	-	-	-	-	50
Older students	4 (24)	16	50	17	17	33	17	17	-	33	-
University lecturers	5 (29)	-	60	20	20	80	20	-	-	-	-
Teachers	8 (47)	22	55	11	11	89	-	-	-	-	11
Sports teams members	4 (24)	50	-	50	-	25	-	-	-	50	25
LGBT society members	1 (6)	100	-	-	-	100	-	-	-	-	-
In the media											
Social media creators	6 (35)	100	-	-	-	83	-	-	-	17	-
Sports people	6 (35)	14	57	14	14	14	43	14	-	14	14
Activists	2 (12)	-	100	-	-	-	66	-	-	-	33
Musicians	7 (41)	-	50	20	30	10	10	20	20	10	20
Scientists	2 (12)	-	50	50	-	-	-	-	-	-	100
Artists	2 (12)	-	100	-	-	-	-	-	50	-	50
Politicians	1 (6)	-	100	-	-	-	-	-	-	-	100
Actors	2 (12)	-	50	50	-	50	-	-	-	-	50
Authors	2 (12)	-	100	-	-	-	25	-	-	25	50
Fictional Characters	8 (47)	10	60	30	-	40	-	-	-	20	40

For gender, F = female, M = male, GMi = gender minority, N/S = not stated and their sexual orientation (L/GW = lesbian/gay woman, GM = gay man, B/P = bisexual/pansexual, Q = queer, H = heterosexual, N/S = not stated).

**Table 3:** Participants’ suggestions about how to incorporate role models into therapy.

Ideas of How to Incorporate into Therapy	Participant
Buddy schemes	4, 6, 10, 12
Compiling lists for users	2, 3, 6, 7, 11, 13, 17
Using role models as a template	5, 7, 8, 9, 12, 15, 16
Comparing role models to me	3, 5
Asking about own role models	1, 2, 5, 6, 9, 17
Physically talking to role models	6, 7, 14
Limitations	
Fixating on role models	3, 5, 6, 7, 8, 9, 11, 14
Feeling inferior to role models	4, 8, 10, 12, 13, 16
Using negative role models	6, 7, 14
Six sessions perceived as too short	8
Conflicting ideas over what a role model is	8
Preference for group therapy over 1-2-1 therapy	12, 15, 16, 17

**Table 4:** Showing places that participants recommended to find role models outside of therapy.

Ideas of How to Incorporate into Therapy	Participant
In Real Life/Personal	
School	1, 3, 6, 10, 12, 17
University	3, 10
Within family	5
LGBTQ+ networks	5, 13
Support groups (incl. mentoring)	1, 10, 14, 17
Hobby groups	4, 16
Volunteering	16
Religious community	4
Non-personal	
Social media	6, 7, 8, 10, 14, 15, 16
Websites	1, 2, 7
YouTube	1
Podcasts	8
TV	1, 8
Books	1, 5, 9, 17

## Data Availability

The datasets presented in this article are not readily available because of privacy and ethical restrictions.
